# Collapsin Response Mediator Proteins: Their Biological Functions and Pathophysiology in Neuronal Development and Regeneration

**DOI:** 10.3389/fncel.2020.00188

**Published:** 2020-06-23

**Authors:** Fumio Nakamura, Toshio Ohshima, Yoshio Goshima

**Affiliations:** ^1^Department of Biochemistry, Tokyo Women’s Medical University, Tokyo, Japan; ^2^Department of Life Science and Medical Bio-Science, Waseda University, Tokyo, Japan; ^3^Department of Molecular Pharmacology and Neurobiology, Yokohama City University Graduate School of Medicine, Yokohama, Japan

**Keywords:** CRMP, neuronal development, regeneration, structural biology, posttranslational modifications, phosphorylation, neurological disorders, drug target

## Abstract

Collapsin response mediator proteins (CRMPs), which consist of five homologous cytosolic proteins, are one of the major phosphoproteins in the developing nervous system. The prominent feature of the CRMP family proteins is a new class of microtubule-associated proteins that play important roles in the whole process of developing the nervous system, such as axon guidance, synapse maturation, cell migration, and even in adult brain function. The CRMP C-terminal region is subjected to posttranslational modifications such as phosphorylation, which, in turn, regulates the interaction between the CRMPs and various kinds of proteins including receptors, ion channels, cytoskeletal proteins, and motor proteins. The gene-knockout of the CRMP family proteins produces different phenotypes, thereby showing distinct roles of all CRMP family proteins. Also, the phenotypic analysis of a non-phosphorylated form of CRMP2-knockin mouse model, and studies of pharmacological responses to CRMP-related drugs suggest that the phosphorylation/dephosphorylation process plays a pivotal role in pathophysiology in neuronal development, regeneration, and neurodegenerative disorders, thus showing CRMPs as promising target molecules for therapeutic intervention.

## Introduction

Cell to cell interactions mediated by extracellular molecules drives numerous physiological processes and helps enable coordinated functioning in neuronal development and regeneration. Extracellular signals are often integrated into complex regulatory networks in which cytoskeletal rearrangement and membrane reorganization are precisely regulated. The growth cone is a characteristic structure at the distal tip of growing axons during development. Growth cones are composed of an actin-rich peripheral domain and a microtubule-rich central domain. At the distal tip of the growth cone, finger-like filopodia and sheet-like lamellipodia extend and retract rapidly as they sense the environmental axon guidance cues around the growth cone.

The first member of the Collapsin Response Mediator Proteins (CRMPs) family, was originally identified as an intracellular protein mediating the action of semaphorin-3A (Sema3A)-signaling, a repulsive axon guidance molecule (Goshima et al., 1995). The initial name of the protein was CRMP-62 because collapsin is the former name of Sema3A and 62 was the new molecule’s molecular weight. As CRMP-62 has significant homology with UNC-33, which is involved in axon guidance in C. elegans through the regulation of tubulin-cytoskeleton, CRMP-62 has been thought to mediate the intracellular signaling involved in axon guidance *via* its modulation of the cytoskeleton at various developmental stages. After the identification of CRMP-62, an additional four members of the CRMP family were identified by several groups, such as TOAD-64, Ulip, DRP, DPYSL (Schmidt and Strittmatter, 2007). Currently, the nomenclature has been unified by calling the family members “CRMP1” through “CRMP5” (Supplementary Table S1); CRMP-62 has been renamed as CRMP2. The CRMP family of proteins are now recognized as multifunctional proteins, not only being involved in neuronal development, regeneration and inflammation, but also in various neurological and psychiatric disease states (Tobe et al., 2017; Tsutiya et al., 2017).

In this review, we summarize the molecular aspects of the CRMPs and discuss their possible involvement in pathophysiological conditions of various disease states. Comprehensive reviews on the implication of CRMPs in Alzheimer’s disease (AD) and psychiatric disorders have been described elsewhere (Gu and Ihara, 2000; Yamashita and Goshima, 2012; Quach et al., 2015; Hensley and Kursula, 2016; Nagai et al., 2017; Tobe et al., 2017; Nakashima et al., 2018).

## Structure of the CRMPs

CRMP1, 2, and 4 have long and short alternate splicing isoforms (Leung et al., 2002). Short isoforms of CRMP1, 2 and 4, CRMP3, and CRMP5 are 565–572 amino acid lengths. The apparent molecular size of these proteins on SDS-PAGE is 62–65 kDa. The long isoforms of CRMP1, 2, and 4 extend approximately 100 amino acids at their N-termini and exhibit 72–75 kDa on SDS-PAGE. We hereafter describe long and short isoforms as “L-CRMP” and “CRMP,” respectively. CRMP1 to CRMP4 share 69–76% amino acid identity while these members and CRMP5 share approximately 50% identity. The long isoforms of CRMPs are minor components in most of the cells and organs. The amino acid identity of the N-terminal regions of L-CRMP1, 2, and 4 is 35% to 54%. The N-terminal extended region has several unique functions such as distal localization of L-CRMP2 (CRMP2A) in axons (Balastik et al., 2015), L-CRMP4 (CRMP4b) and RhoA interaction in Nogo signaling (Alabed et al., 2007), and correlation of L-CRMP1 expression and cancer cell migration (Pan et al., 2011).

**Figure 1:**
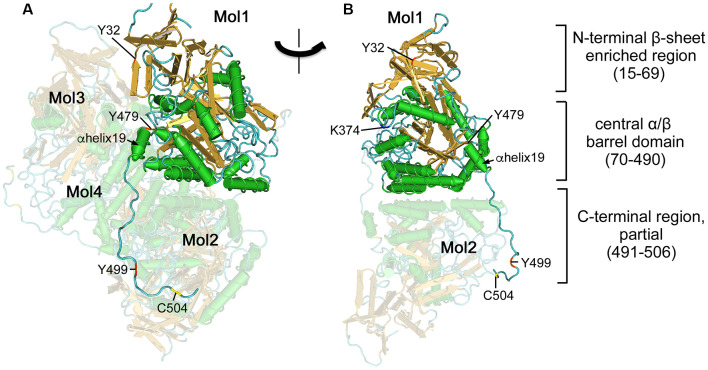
Ternary structure of Collapsin response mediator proteins 2 (CRMP2). **(A)** Crystal structure of human CRMP2 (1–525) homo-tetramer (5X1A; Niwa et al., 2017). A secondary structure-based view was rendered using Cn3D (version 4.3). One monomer (Mol1) is shown in secondary structure-based color (α-helix, green; β-sheet, gold; random coil, cyan). The structure is visible between 14 and 506 residues. Phosphorylation (Tyr32, Tyr479, and Tyr499), SUMOylation (Lys374), and oxidation (Cys504) sites are indicated by red, blue, and yellow, respectively. Other monomers (Mol2–4) are shown in faded colors. The C-terminal domain of Mol1 interacts with Mol2. **(B)** Rotate view. The interface of Mol1 to Mol3 is shown by omitting Mol3 and Mol4. Note that Lys374 and Tyr479 are present on the interface.

X-ray crystal structures of the short isoforms of CRMP1, 2, 4, and 5 have been reported (Deo et al., 2004; Stenmark et al., 2007; Ponnusamy and Lohkamp, 2013; Ponnusamy et al., 2014). Central regions of the CRMPs (8–490) forms a tetramer (Figure 1). The folded CRMP structure resembles dihydropyrimidinase (DHPase), which hydrolyzes the amide bond of pyrimidine bases (Gojkovic et al., 2003). Each CRMP monomer consists of an N-terminal β-sheet enriched region (15–69) and central α/β barrel domain (70–490). The central domain contains tetramer interfaces. The ternary structure of the entire CRMP C-terminal region (490–572) has not been determined possibly because the region stays flexible and the structure is somewhat random, altering its conformation upon posttranslational modification such as phosphorylation. However, the partial ternary structure of the C-terminal proximal region of CRMP2 has been reported (Niwa et al., 2017). The C-terminal visible residues from the α-helix19 (480–487) further extend in the same direction and several residues in (491–506) interact with the neighboring monomer (Figure 1), contributing to stabilizing the tetramer. It has been shown that CRMPs form hetero-oligomerized complexed in the brain (Wang and Strittmatter, 1996). *In vitro* reconstitution revealed that CRMP1, CRMP2, and CRMP3 prefer hetero oligomerization. However, the biological significance of the hetero-complexed CRMPs has not yet been fully addressed (Makihara et al., 2016).

Although the CRMPs have 51–54% amino acid identity with DHPase, they have no enzymatic activity toward dihydropyrimidines (Wang and Strittmatter, 1996), as His69 and His248 residues coordinating Zn ions in the catalytic center of DHPase are not conserved in the CRMPs. Instead, CRMP3 has histone H4 deacetylase activity, which is involved in neuronal cell death after the translocation of CRMP3 to the nucleus (Hou et al., 2013). Other enzymatic activities of the CRMPs are currently unknown.

## Phosphorylation of CRMPs

**Figure 2:**
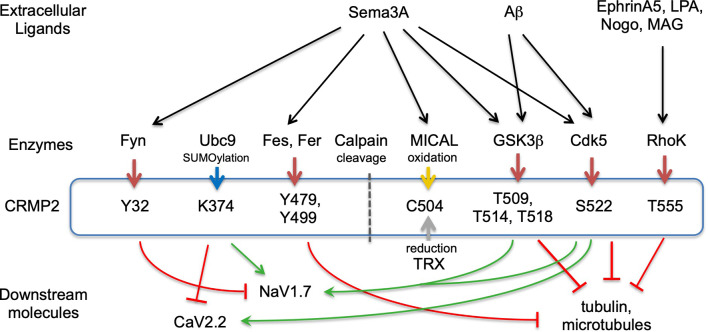
CRMP2-posttranslational modification and signaling. Extracellular ligands, modulating enzymes, posttranslational medication sites in CRMP2, and downstream molecules are represented. Red arrows from enzymes to CRMP2 represent phosphorylation. Blue, yellow, and gray arrows indicate SUMOylation, oxidation, and reduction, respectively. Green and red stop arrows represent facilitation and inhibition, respectively. Not all ligands or downstream molecules are shown.

Posttranslational modifications play a crucial role in regulating the folding of proteins, their targeting to specific subcellular compartments, their interaction with other proteins, and their functional states, such as activation and inactivation in signal transduction pathways. Protein phosphorylation is the major molecular mechanism through which protein function is regulated in response to extracellular stimuli both inside and outside the nervous system. Many kinases phosphorylate CRMPs (Figure 2, Supplementary Table S2). Most of the phosphorylation sites are localized in their C-terminal regions. As the primary structures of their C-termini are relatively not conserved compared to their central domains, some kinases specifically phosphorylate one of the CRMPs but not the others. Such phosphorylation may contribute to the unique function of each member of the CRMP family protein. Here we summarize the phosphorylation by kinase-based classification.

Cyclin-dependent kinase 5 (Cdk5) and glycogen kinase 3β (GSK3β)—the phosphorylation of CRMP2 by Cdk5 and GSK3β is the most extensively studied among the CRMP members (Figure 2). CRMP2 is primarily phosphorylated at Ser522 by Cdk5 and subsequently phosphorylated at Ser518, Thr514, and Thr509 by GSK3β (Cole et al., 2006; Brittain et al., 2012; Yamashita and Goshima, 2012; Nagai et al., 2017). The latter phosphorylation requires Cdk5-primed phosphorylation of Ser522, therefore, the substitution of Ser522 with Ala eliminates the phosphorylation of Ser518, Thr514, and Thr509 in CRMP2. CRMP’s phosphorylation state determines its biological function. Non-phosphorylated CRMP2 binds tubulin dimers to facilitate the axonal elongation; the phosphorylation eliminates that function. Instead, the phosphorylated CRMP2 acts as an intracellular signaling mediator for inhibition of axonal guidance such as *via* Sema3A. Recent crystal structural analysis of CRMP2 revealed that non-phosphorylated CRMP2 monomer forms hetero-trimer between CRMP2 monomer and the GTP-tubulin hetero-dimer (Sumi et al., 2018). Using the phosphorylation-mimicking form of CRMP2, Sumi et al. (2018) revealed that the increased negative charge of the C-terminal region alters CRMP2 homo-tetramer conformation and reduces the interaction of CRMP2 and tubulin-dimers. Ser522 phosphorylation of CRMP2 augments the interaction with CaV2.2 to enhance Ca^2+^ influx (Brittain et al., 2012).

Phosphorylation state is not only regulated by kinase but also by phosphatases that dephosphorylate. PP2A dephosphorylates CRMP2 Thr514 and facilitates the non-phospho CRMP2 effect on neurite outgrowth (Zhu et al., 2010). L-CRMP2 is phosphorylated by Cdk5 at Ser27 in N-terminal extended region as well as at Ser623, an equivalent residue of Ser522 in the short form (Balastik et al., 2015). Prolyl Isomerase Pin1 catalyzes and stabilizes phosho-Ser32-Pro33 in L-CRMP2. This brings distal localization of L-CRMP2 in axons and attenuates Sema3A-repulsive response despite Cdk5-dependent phosphorylation.

The C-terminal phosphorylation sites, Thr509, Thr514, Ser518, and Ser522, are conserved in human CRMP1 and CRMP4. Different priming kinases, Cdk5 and dual-specificity tyrosine phosphorylation-regulated kinase 2 (DYRK2) phosphorylate Ser522 of CRMP1 and CRMP4, respectively (Cole et al., 2006). GSK3β secondarily phosphorylates human CRMP1 and CRMP4 like CRMP2. However, Cdk5 directly phosphorylates Thr509 of mouse/rat CRMP1 because Thr514 is replaced with Ala. CRMP5 is phosphorylated by GSK3β at Thr516 and this phosphorylation is essential for tubulin-binding of CRMP5 but inhibits neurite outgrowth (Brot et al., 2014).

Rho-kinase—Rho-kinase phosphorylation of Thr555 CRMP2 was initially identified by *in vitro* kinase assays (Arimura et al., 2000). As Rho-kinase acts as the downstream molecule of lysophosphatidic acid (LPA), the primary cultured dorsal root ganglion (DRG) neurons stimulated with LPA induced CRMP2-Thr555 phosphorylation (Figure 2). Overexpression of CRMP2-Thr555Ala in DRG neurons suppressed the LPA-induced growth cone collapse response but not the Sema3A-response. A similar mechanism is utilized in Ephrin-A5 signaling. Ephrin-A5-stimulation induced growth cone collapse response and the Thr555 phosphorylation in cultured DRG neurons. This response was abrogated by a Rho-kinase inhibitor or by the overexpression of CRMP2 Thr555Ala (Arimura et al., 2005).

Other Ser/Thr kinases—it has been shown that Protein kinase A (PKA), Protein kinase C (PKC), and Calmodulin kinase II (CaMKII) phosphorylate CRMP2 in pathological conditions. PKA-dependent phosphorylation of CRMP2 was observed in the nucleus accumbens neurons of cocaine-sensitized rats (Boudreau et al., 2009). The PKA-phosphorylation site has not been determined. PKC-βII phosphorylates CRMP2 at the Thr514 residue. This phosphorylation prevents calpain-mediated CRMP2 proteolysis during ischemic injury (Yang et al., 2016). CaMKII phosphorylates CRMP2 at Thr555, which, in turn, attenuates glutamate toxicity in ischemic brains (Hou et al., 2009).

Tyrosine kinases—it has been shown that the constitutive active form of Fyn phosphorylates all members of CRMPs (Uchida et al., 2009). The constitutive active form phosphorylates Tyr32 of CRMP2; this phosphorylation is involved in Sema3A-signaling in mouse DRG neurons (Figure 2). In contrast, wild-type Fyn selectively phosphorylates CRMP1 at Tyr504 but not other CRMPs (Buel et al., 2010). The absence of an autoinhibitory mechanism in the constitutive active form may contribute to the non-selective phosphorylation of CRMPs. Fyn-related kinase Fes and Fer phosphorylate CRMP2 and CRMP5. *In vitro* phosphorylation assay and mass spectrometry analysis revealed that Fer phosphorylates CRMP2 at the 32, 251, 275, 431, 479, and 499 Tyr residues (Zheng et al., 2018). Among these sites, Tyr479 and/or Tyr499 phosphorylation prevents the tetramerization of CRMP2, which, in turn, decreases the interaction of CRMP2 and microtubules (Figures 1, 2).

## Other Posttranslational Modifications

SUMOylation—CRMP2 is post-translationally modulated by a small ubiquitin-like modifier (SUMO) at Lys374, and this modulation alters CRMP2-interaction with Ca^2+^ and Na^+^ channels in a different manner (Figure 2). While SUMOylated CRMP2 enhances Na^+^ currents through NaV1.7 surface expression (Dustrude et al., 2013), it reduces Ca^2+^ influx through CaV2.2 (Ju et al., 2013). De-SUMOylation and de-phosphorylation of CRMP2 at Thr514 contribute to the formation and maturation of dendritic spines (Zhang et al., 2018). As the SUMO consensus motif including Lys374 is conserved among CRMPs, SUMOylation may regulate other CRMPs.

Oxidation—genetic dissection of drosophila axon guidance revealed the involvement of flavoprotein oxidoreductase MICAL in semaphorin-signaling (Terman et al., 2002). CRMP2 binds and activates MICAL, by releasing its autoinhibition domain (Schmidt et al., 2008). CRMP2 is subsequently oxidized at Cys504 by MICAL upon Sema3A-stimulation and transiently forms disulfate bonds with thioredoxin (TRX; Figure 2). The CRMP2-TRX complex facilitates the phosphorylation of CRMP2 at Thr509 by GSK3β (Morinaka et al., 2011), indicating cross-talk between oxidation and phosphorylation in Sema3A-signaling.

Proteolysis—various brain injuries such as ischemia and glutamate toxicity induce calpain-mediated cleavage of all members of the CRMP family and produce approximately 54 kDa truncated products (Jiang et al., 2007). Calpain cleavage sites have been identified in CRMP3 and CRMP4. CRMP3 is cleaved at N-terminal Arg75-Leu76 bond and CRMP4 is at the C-terminal Arg550-Ser551 bond (Kowara et al., 2005; Hou et al., 2006). Processing of CRMP2 at the C-terminal region exposes nuclear localization signal (NLS) within residues Arg471-Lys472, which brings the truncated CRMP2 to the nucleus (Rogemond et al., 2008). Each truncated CRMP has a unique function. Truncated CRMP2 suppresses neurite outgrowth and reduces surface expression of the NR2B NMDA receptor subunit to protect neurons from glutamate toxicity (Bretin et al., 2006; Rogemond et al., 2008). Cleaved CRMP3 translocates to the nucleus and acts as a histone H4 deacetylase, which, in turn, induces neuronal cell death (Hou et al., 2006; Hou et al., 2013).

## CRMP Interacting Molecules

CRMPs interact with membrane and intracellular proteins (Supplementary Table S3). Here, we classify those proteins into several categories including cytoskeletal proteins and ion channels.

Tubulin and microtubules—overexpression CRMP2 in hippocampal neurons switch neurite identity from dendrites to axons (Fukata et al., 2002; Yoshimura et al., 2005). This action is accomplished by two functions (Niwa et al., 2017). First, CRMP2 monomers bind to tubulin dimers and transport them to the distal end of axons to facilitate the neurite outgrowth. Second, CRMP2 tetramer stabilizes microtubules (Lin et al., 2011). These actions are canceled by the C-terminal phosphorylation of CRMP2 by GSK3β because phosphorylated CRMP2 loses its binding ability (Figure 2). It has been shown that Fer phosphorylates CRMP2 at Tyr479 and Tyr499. Crystal structures of the CRMP2-Tyr479Glu phospho-mimicking mutant revealed that the phosphorylation of Tyr479 interferes with the tetramerization of CRMP2 by introducing a negative charge in the hydrophobic cavity of the tetramer interface (Zheng et al., 2018). CRMP2 α-helix 19 (476–487) serves as a tubulin-dimer interface (Niwa et al., 2017). As CRMP2-tubulin dimer interaction is abrogated by the C-terminal phosphorylation of CRMP2 (Sumi et al., 2018), this modulation would also alter the higher structure of the C-terminal region and α-helix 19. However, such alteration of full-length CRMPs has not been revealed in a structural basis. CRMP3 inhibits tubulin polymerization and neurite outgrowth in cultured cerebellar granule neurons (Aylsworth et al., 2009). CRMP4 interacts with microtubules in hippocampal neurons to facilitate axon outgrowth (Khazaei et al., 2014). CRMP5 is phosphorylated by GSK3β at Thr516 (Brot et al., 2014). This phosphorylation is essential for tubulin-binding of CRMP5 but inhibits neurite outgrowth.

Actin—CRMP1 indirectly regulates actin-cytoskeleton through the interaction with actin-binding proteins including filamin-A and Ena/VASP proteins (Nakamura et al., 2014; Yu-Kemp et al., 2017). Interestingly, distinct roles were demonstrated for CRMP1 and CRMP2 in steering axonal outgrowth, using microscale-chromophore-assisted local inactivation (micro-CALI) method (Higurashi et al., 2012). CRMP1 and CRMP2 have characteristic distribution in the growth cones. CRMP1 was co-localized with actin in the peripheral domain, while CRMP2 was co-localized with tubulin in the central domain. It has been shown that CRMP4 binds F-actin and causes actin bundling (Rosslenbroich et al., 2005). This actin bundling is involved in the filopodia extension of hippocampal growth cones (Khazaei et al., 2014). CRMP5 interacts with actin and tubulin in growth cones (Gong et al., 2016). CRMP5 is localized in filopodia of growth cones and its overexpression promotes filopodial formation (Hotta et al., 2005).

Motor proteins—both Antero- and retro-grade motor proteins bind to CRMP2. Kinesin-1 binds to CRMP2 through the interaction of the kinesin light chain and the C-terminal region of CRMP2 (Kimura et al., 2005). In contrast, the N-terminus of CRMP2 binds to the dynein heavy chain but the interaction interferes with the retrograde transporting activity (Arimura et al., 2009). Combining these actions may facilitate the transport of the CRMP2-tubulin dimer complex to the distal end of the axons. The CRMP2 and Kinesin-1 complex also transports TrkB-containing vesicles to axonal plasma membranes (Arimura et al., 2009).

Na^+^ channel—CRMP1 interacts with NaV1.7 and modulates the Na^+^ currents by lowering the threshold of the channel (Yamane et al., 2017). This action is probably augmented by C-terminal phosphorylation because the overexpression of phosphomimetic CRMP1-Thr509Asp/Ser522Asp mutant enhanced the current. CRMP2 also interacts with NaV1.7 and phosphorylation of Thr509 and Ser522 of CRMP2 augments the action (Dustrude et al., 2013). This interaction is modulated by SUMOylation and phosphorylation of CRMP2. While Ser522 phosphorylation and Lys374 SUMOylation prevent NaV1.7 internalization, Tyr32-phosphorylation facilitates the endocytosis of NaV1.7 (Dustrude et al., 2016).

Ca^2+^ channel—CRMP2 binds to CaV2.2 and increases Ca^2+^ current by cell surface expression of the channel (Brittain et al., 2009). Cdk5-phosphorylation at Ser522 augments the interaction with and Ca^2+^ influx through CaV2.2 (Brittain et al., 2012). However, in contrast to NaV1.7 modulation, Lys374 SUMOylation of CRMP2 attenuates the Ca^2+^ current (Ju et al., 2013). Three domains in CRMP2, CBD1(94–166), CBD2(212–297), and CBD3(479–500), are involved in CaV2.2-binding (Brittain et al., 2009). The peptide fuses TAT (transduction domain of HIV), and CBD3 interferes with CRMP2 and CaV2.2 coupling to suppress inflammatory and neuropathic pain in model mice (Brittain et al., 2011). This effect is due to the reduction of membrane surface expression of CaV2.2. As the CBD3 sequence partially overlaps with α-helix 19 (476–487), a tubulin-dimer binding domain, TAT-CBD3 may also affect CRMP2-tubulin interaction. Also, CRMP2 interacts with NMDAR and Na/Ca Exchanger 3 to facilitate glutamate-induced Ca^2+^ dysregulation in hippocampal neurons (Brustovetsky et al., 2014). CRMP3 facilitates the depolarization-evoked Ca^2+^ response of L- and N-type Ca channels to promote dendrite morphogenesis of hippocampal neurons (Quach et al., 2013).

Other membrane and intracellular proteins—CRMP1 interacts with actin-binding protein Filamin-A. This interaction is augmented by the Cdk5-phosphorylation of CRMP1 (Nakamura et al., 2014). CRMP1-binding to Filamin-A decreases the F-actin gelation cross-linked by Filamin-A, which, in turn, facilitates remodeling of the actin cytoskeleton. CRMP1 also interacts with EVL, one of Ena/VASP proteins, to facilitate actin filament elongation at the barbed end (Yu-Kemp et al., 2017). CRMP2 interacts with Numb, one of the endocytosis adaptor proteins, to regulate endocytosis and recycling of axonal growth cone membranes (Nishimura et al., 2003). Long-form CRMP4, but not short-form, interacts with RhoA and suppresses neurite outgrowth (Alabed et al., 2007). GSK3β phosphorylates L-CRMP4 at Thr622 and attenuates RhoA-binding and cancels the suppression (Alabed et al., 2010). Myelin-associated inhibitors (MAIs) bring their inhibitory action through the inactivation of GSK3β and dephosphorylation of L-CRMP4 Thr622. Other known interactions are summarized in Supplementary Table S3.

## Neuronal Subcellular Localization of CRMPs

Each CRMP has a different subcellular localization in a developmental-stage dependent manner. In primary cultured cortical neurons, CRMP1 and CRMP2 are predominantly expressed in axons and, to a lesser extent, in the somatodendritic regions at 7 days *in vitro* (DIV 7; Makihara et al., 2016). By DIV14, while CRMP1 tends to localize to the presynaptic region, CRMP2 distributes to the axons and dendrites.

Alternative splicing also contributes to localization. L-CRMP2 localizes to the distal axons of hippocampal neurons (Balastik et al., 2015). Studies in mice in which CRMP3 has been knocked-out revealed that CRMP3 regulates the dendritic growth of hippocampal neurons (Quach et al., 2013). CRMP4 distributes along axons and dendrites of hippocampal neurons and facilitates their growth through its interaction with actin and tubulin (Khazaei et al., 2014; Cha et al., 2016). CRMP5 is involved in the dendritic growth of hippocampal neurons (Yamashita et al., 2011). The effect of CRMP5 on axon outgrowth is inconsistent i.e., it can both variably promote and inhibit it (Brot et al., 2014; Gong et al., 2016); however, the inhibitory effect may reflect Thr516 phosphorylation of CRMP5 by GSK3β (Brot et al., 2014).

The neuronal growth cone consists of an actin-rich peripheral region and a tubulin-rich central domain. The peripheral region is further divided into filopodia and lamellipodia. CRMP1 localizes to both the peripheral and central domains, while CRMP2 localizes to the central domain in chick DRG growth cones (Higurashi et al., 2012). Focal inactivation of CRMP2 by micro-CALI support the role of CRMP2 in microtubule extension and stabilization. CRMP4 localizes to both the peripheral and central regions of hippocampal growth cones (Khazaei et al., 2014). Peripheral and central CRMP4 participate in filopodial F-actin bundling and microtubule elongation, respectively. CRMP5 predominantly localizes to the peripheral region of hippocampal growth cones to promote filopodial formation (Hotta et al., 2005).

Nuclear-translocation of calpain-digested CRMPs has already been discussed above under the “Proteolysis” section.

## The Relation of CRMPs and Axon Guidance Molecules

Sema3A, a prototypical inhibitory axonal guidance molecule, regulates axonal projection of various neurons in the peripheral and central nervous system (CNS; Nakamura et al., 2000). It also participates in the dendritic growth and synapse formation and maturation (Goshima et al., 2016). The role of the CRMPs in Sema3A-signaling has been studied both *in vitro* and *in vivo*. Sema3A binds the NRP1 and Plexin-A receptor complex, which activates at least three distinct downstream signaling pathways; the phosphorylation cascade, the small-G-protein cascade, and MICAL-mediated oxidation. CRMPs are involved in all of these signaling pathways.

Sema3A activates Cdk5 and GSK3β kinases, which sequentially phosphorylates CRMP2 Ser522 and Thr509 residues, respectively. Phosphorylation of CRMP2 at Tyr32 by Fyn or Fes has also been shown to be involved in Sema3A-signaling (Yamashita et al., 2012; Figure 2). Phosphorylation makes CRMP2 alter its binding partners from tubulin-dimer to other downstream molecules such as CaV2.2 (Brittain et al., 2012). CRMP1 also acts on molecules downstream of Sema3A by being phosphorylated at Ser522 by Cdk5 (Yamashita et al., 2012). CRMP2 binds and activates α2-chimaerin, a Rac-GTPase activating protein, to downregulate Rac. This action is involved in the Sema3A response in DRG neurons (Brown et al., 2004). CRMP2 binds and activates MICAL by releasing its autoinhibition domain (Schmidt et al., 2008).

*Sema3a*-knockout mice demonstrate that Sema3A restricts peripheral neuron projections while facilitating the formation of dendrites and synapses in CNS. *Sema3a*^−/−^ mice showed an overshooting and defasciculation of trigeminal branches and DRG neurons (Taniguchi et al., 1997). *Crmp2*^−/−^ mice show CRMP2 to be involved in the peripheral projection of the trigeminal ophthalmic branches (Ziak et al., 2020). However, as the irregular peripheral projection of DRG axons is absent in *Crmp2*^−/−^ mice, CRMP2 may mediate additional guidance cues such as ephrin-A5 and Slit2 in this projection (Jayasena et al., 2005; Kubilus and Linsenmayer, 2010).

The involvement of CRMPs in Sema3A-signaling is more evident in CNS development. Synapse formation of cortical pyramidal neurons is reduced in *Sema3a^−/−^*, *Crmp2*^−/−^, and *Crmp2*^−/−^ mutants compared to wild-type (Makihara et al., 2016). *Sema3a*^+/–^; *Crmp1*^+/–^ and *Sema3a*^+/–^; *Crmp2*^+/–^ double heterozygous mice exhibit a similar phenotype, indicating that both CRMP1 and CRMP2 act as downstream molecules of Sema3A-regulated synapse formation. However, as *Crmp1*^+/–^; *Crmp2*^+/–^ double heterozygous showed normal phenotype, CRMP1 and CRMP2 may mediate different intracellular signaling pathways.

Considering the obvious role of CRMPs in synapse maturation, it is noteworthy that the “lithium-response (LiR) pathway” governs the phosphorylation state of CRMP2, hence yielding insight into the pathogenesis of bipolar disorder (BPD; Tobe et al., 2017). Proteomic and phospho-proteomic analysis of human-induced pluripotent stem cells (hiPSCs) and their neuronal derivatives showed that the “set-point” for the ratio of inactive phosphorylated CRMP2 to active non-phosphorylated CRMP2 is elevated uniquely in LiR BPD patients, but not in patients with other psychiatric (including lithium-nonresponsive BPD) or neurological disorders. Lithium (and other CRMP2 pathway modulators) lowers pCRMP2, increasing dendritic spine area and density. Actual human BPD brains show similarly elevated CRMP2 ratios and diminished spine densities; lithium therapy normalizes the ratios and spines. Behaviorally, transgenic mice that reproduce lithium’s postulated site-of-action in dephosphorylating CRMP2 emulate lithium responsiveness in BPD.

The CRMPs also play roles as downstream target genes of signaling of other axon guidance molecules, morphogens, and cytokines in neuronal development and function. BMP-Smad1 signal suppresses CRMP2 gene expression (Sun et al., 2010). TGF-β signaling regulates neuronal morphogenesis through the suppression of CRMP2 expression during brain development (Nakashima et al., 2018). Nicotine administration causes gene up-regulation of CRMP2 in adult mice during nicotine-induced hippocampal long-term-potentiation (Kadoyama et al., 2015).

Sema3F binds Neuropilin-2 (NRP2)/Plexin-A complex and activates intracellular signaling like Sema3A. *Sema3f* and *Nrp2* knockout mice showed axon guidance defects including disorganized anterior commissure and pruning defect of hippocampal mossy fiber axons in the infrapyramidal bundle (Chen et al., 2000; Sahay et al., 2003). *Crmp2*^−/−^ mice exhibit reduction of the corpus callosum, hypoplastic anterior commissure, and defective pruning of CA3 infrapyramidal bundle and corticospinal visual axons (Ziak et al., 2020). As *Crmp2*^−/−^ mice show normal pruning of the hippocampo-septal bundle, of which projection is regulated by Sema3A, CRMP2 is involved in Sema3F-regulated axon guidance rather than Sema3A-guidance.

Reelin regulates neuronal cell migration including neocortex six-layer formation and hippocampal lamination (Yamamoto et al., 2009). CRMP1 is involved in Reelin signaling (Yamashita et al., 2006). Radial migration of cortical neurons is delayed in *Crmp2*^−/−^ brain. Dab1, an adaptor protein of Reelin signaling, is co-localized with CRMP1 in migrating neurons. Homozygous Dab1 yotari mutant mice, *Dab*1^ y*ot/yot*^, exhibit disrupted hippocampal lamination. A similar phenotype is observed in *Crmp2*^−/−^; *Dab1*^+/yot^ mice but not in *Crmp2*^−/−^ or *Dab1*^+/yot^ mutants. This genetic augmentation suggests that CRMP1 is involved in the Reelin-regulated neuronal cell migration.

Nogo, an inhibitory signal for neurite outgrowth after spinal cord injury (SCI), exerts the action through NgR1 and its associated transmembrane proteins. It has been shown that CRMP2 is involved in the downstream of Nogo-signaling by the Rho-kinase phosphorylation at Thr555 (Mimura et al., 2006; Petratos et al., 2012). As Nogo downstream signaling, L-CRMP4 (CRMP4b) binds to RhoA and interferes with neurite outgrowth (Alabed et al., 2007). NgR1 forms a receptor complex with Plexin-A2, which associates with CRMP2 and CRMP4 in Nogo dependent manner (Sekine et al., 2019).

Repulsive guidance molecule-a (RGMa) exerts its inhibition through the phosphorylation of CRMP2 by Rho-kinase and GSK3β (Wang et al., 2013). Chondroitin sulfate proteoglycan (CSPG) is a major inhibitor of axonal regeneration after CNS trauma. Knockout mice studies revealed that CRMP4 mediates the action of CSPG and/or other inhibitory molecules related to SCI (Nagai et al., 2015, 2016). The functional recovery of motor and sensory neurons from SCI is accelerated in *Crmp4*^−/−^ mice. Sema4D binds Plexin-B1 and downregulates R-Ras and PI3K-Akt signaling. This brings the activation of GSK3β and CRMP2 phosphorylation at Thr514 (Ito et al., 2006). Plexin-A also inactivates R-Ras by the same mechanism in Sema3A-signaling. *In vivo* Sema4D-CRMP2 relation has yet to be examined.

## CRMPs in CNS Regeneration and Degeneration

After nerve injury, peripheral nervous system (PNS) axons form growth cones and regenerate (Ertürk et al., 2007). However, axons in the CNS fail to form tips and instead become dystrophic retraction bulbs (Ertürk et al., 2007; Bradke et al., 2012). Growth cones contain organized microtubules that form tight bundles along with axonal axis, whereas retraction bulbs have disassembled microtubules. The importance of the microtubule state for growth cone structure is supported by the fact that the application of the microtubule-destabilizing agent nocodazole transforms the growth cone into a retraction bulb-like structure *in vitro*, resulting in axonal growth arrest (Ertürk et al., 2007).

Phosphorylation of CRMP2 at its C-terminal domain by serine/threonine (Ser/Thr) kinases causes growth cone collapse *via* microtubule destabilization, while inhibition of C-terminal phosphorylation stabilizes the microtubules (Yamashita and Goshima, 2012; Nagai et al., 2017). CRMP2 phosphorylation is also induced during Wallerian degeneration after optic nerve injury. During Wallerian degradation, zinc/RING finger protein 1 (ZNRF1)-dependent protein kinase B (Akt) degradation increases GSK3β activity and results in an increase in CRMP2 phosphorylation at Thr514 (Wakatsuki et al., 2011). The introduction of the CRMP2-Thr514Ala (CRMP2^T514A^) virus suppresses the Wallerian degradation of the optic nerve *in vivo* (Wakatsuki et al., 2011). To delineate the *in vivo* role of CRMP2 phosphorylation at Ser522, the CRMP2^S522A/S522A^ (CRMP2^KI/KI^) mouse was generated (Yamashita et al., 2012). Due to the phosphorylation of Ser522 by Cdk5 functions, as priming phosphorylation followed GSK3β phosphorylation of Thr509/Thr514/Ser518, 4 sites of phosphorylation in the C-terminal domain of CRMP2 are eliminated in the CRMP2^KI/KI^ mice (Yamashita et al., 2012). Wallerian degeneration following optic nerve injury was found to be suppressed in CRMP2^KI/KI^ mice (Kinoshita et al., 2019). Axonal damage of the optic nerve induces retinal ganglion cell death in the wild-type mice (Duan et al., 2015). However, suppression of retinal ganglion cell death is observed in CRMP2^KI/KI^ mice (Kondo et al., 2019). Moreover, axonal regeneration was promoted in CRMP2^KI/KI^ mice. CRMP2^KI/KI^ mice showed higher expression of the regenerative axonal marker growth-associated protein 43 (GAP43). Tracing of the axon by injecting an anterograde tracer into the eye showed elongated optic nerves in CRMP2^KI/KI^ mice, while labeled axons were limited in wild-type mice after optic nerve injury. These results are in agreement with the observation that optic nerve regeneration occurs after the intravitreal administration of the CRMP2^T514A^ virus (Leibinger et al., 2017). Overexpression of wild-type CRMP2 *via* a viral infection promoted regeneration of the hypoglossal nerve in the adult rats (Suzuki et al., 2003). In an SCI model, microtubule-stabilizing agents have been found to promote functional recovery and serotonergic axon regeneration (Hellal et al., 2011). CRMP2^KI/KI^ mice also showed better functional, motor and sensory recovery, and serotonergic axon regeneration (Nagai et al., 2016). CRMP2^KI/KI^ DRG neurons showed an enhanced neurotrophic response to brain-derived neurotrophic factor and a hindered inhibitory response against CSPG. These alternations, which were in response to external factors, may also be linked to the recovery in CRMP2^KI/KI^ mice after SCI.

Recently, the mutant superoxide dismutase (SOD)1^G93A^ mouse model of amyotrophic lateral sclerosis (ALS) was crossed with the CRMP2^KI/KI^ mice to assess the genetic inhibition of CRMP2 phosphorylation. Compared to baseline, CRMP2^KI/KI^ mice x SOD1^G93A^ mice developed a slower degeneration of axons and neuromuscular junctions and a delayed progression of motor symptoms (Numata-Uematsu et al., 2019). The myelin oligodendrocyte glycoprotein-induced experimental autoimmune encephalomyelitis (EAE) mouse model of multiple sclerosis (MS) showed an increase in CRMP2 phosphorylation at Thr555 in a Nogo-dependent manner (Petratos et al., 2012). The anti-Nogo-A antibody prevented the development of EAE and increased CRMP2 phosphorylation at Thr555. A recent study further demonstrated that abrogation of the NgR1/pCRMP2 signaling cascade maintains Kinesin-1-dependent anterograde axonal transport to limit the inflammation-mediated axonopathy and demyelination of the EAE model (Lee et al., 2019).

Methyl-phenyl-tetrahydropyridine (MPTP) readily penetrates the blood-brain barrier and enters the brain where it is converted into 1-methyl-4-phenylpyridinium (MPP^+^) by MAO-B in astrocytes. MPP^+^ is transported by the dopamine (DA) transporter into DA nerve terminals, and it destroys dopaminergic neurons, thereby causing the symptoms similar to those of Parkinson’s disease (PD). MPTP administration in non-human primates and aged rodents are often used to model PD. The elevation of CRMP2 phosphorylation at Thr514 through Akt/GSK3β was reported in an MPP^+^-PD model in dopaminergic neurons *in vitro* (Fang et al., 2015). Elevation of CRMP2 phosphorylation at Ser522 was observed in dopaminergic neurons in the substantia nigra compacta (SNc) *in vivo* (Togashi et al., 2019). Production of p25 and elevation of Cdk5 activity have been reported in an MPTP-induced PD mouse model (Smith et al., 2003; Cheung and Ip, 2012). Therefore, increased phosphorylation of CRMP2 at Ser522 is consistent with these studies. CRMP2^KI/KI^ mice showed increased axonal viability in the nigro-striatal pathway in an MPTP-induced PD model (Togashi et al., 2019).

Another member of the CRMPs, CRMP4, is reported to be involved in the signal pathway of myelin-associated inhibitors (MAIs; Alabed et al., 2007, 2010; Nagai et al., 2015). MAIs activate ras homolog family member A (RhoA), which interacts with L-CRMP4 to inhibit axonal growth *in vitro*. CRMP4 also interacts with CSPG through the NgR/GSK3β pathway. After SCI, CRMP4^−/−^ mice showed motor and sensory axonal growth (Nagai et al., 2015, 2016). Furthermore, the deletion of CRMP4 prevents DA neuronal loss in SNc and increases axonal viability in the DA neurons in the striatum (Tonouchi et al., 2016).

## The Role of CRMPs in Inflammatory Cells and Glia

In SCIs, scar formation occurs at the lesion site where astrocytes are recruited by the pro-inflammatory cytokines secreted from the activated microglia/macrophages. The compaction and seclusion of infiltrating inflammatory cells in the lesion center occur during the sub-acute phase of SCI. It is generally accepted that glial scars inhibit axonal growth, by physically interacting with the distal tip of axons (Filous et al., 2014) or by secreting extracellular matrix molecules such as CSPGs (Tan et al., 2011; Burnside and Bradbury, 2014; Cregg et al., 2014) and inhibitory axon guidance molecules such as Sema3A (Kaneko et al., 2006).

A reduction in scar formation after SCI in CRMP2^KI/KI^ mice has been reported (Nagai et al., 2015). In rat SCI, activation of GSK3β in reactive astrocytes is involved in the infiltration of inflammatory cells and scar formation by Wnt signaling-mediated β1-integrin expression (Renault-Mihara et al., 2011). Compaction of scar formation was observed in the GSK3β inhibitor treatment of SCI. Therefore, one possible explanation for the reduced scar formation in CRMP2^KI/KI^ after SCI is that it is mediated by the inhibition of CRMP2 phosphorylation.

After SCI, CRMP4 expression is reported in activated microglia/macrophages (Nagai et al., 2015). In cultured BV-2 microglial cells, microglia express CRMP4 after lipopolysaccharide stimulation (Manivannan et al., 2013). CRMP4 is involved in the association of F-actin, cytokine release, migration, and phagocytosis in BV-2 cells. Injection of Zymosan, macrophage-activating agent, to the spinal cord of *Crmp4*^−/−^ mice, showed reduced microglial activation (Nagai et al., 2015). In *Crmp4*^−/−^ mice with SCIs, reduction of scar formation promoted axonal growth and significant locomotor recovery was observed.

Inflammatory responses have been implicated in other causes of neuronal degeneration, including PD. Disease progression has been linked to the secretion of inflammatory cytokines that engage neighboring cells, including astrocytes, which, in turn, induce autocrine and paracrine responses that amplify the inflammation, leading to further neurodegeneration (Niranjan, 2014). Reduced inflammatory response and suppressed DA neuron death after MPTP injection have been observed in CRMP4^−/−^ mice (Tonouchi et al., 2016). Since cell death of DA neurons by MPTP injection was suppressed, the reduced inflammatory response may be a secondary consequence of the limited release of factors from dying neurons. MS is a chronic inflammatory, demyelinating, and neurodegenerative disorder of the CNS. In an EAE mouse model of MS, the importance of CRMP2 Ser522 phosphorylation was demonstrated using CRMP2^KI/KI^ mice (Moutal et al., 2019). CRMP2 is also expressed in the immune system and plays a critical role in T lymphocyte polarization and migration (Vincent et al., 2005). C-X-C motif chemokine 12 (CXCL12)/SDF1 treatment activates T lymphocyte migration by switching the dephosphorylation of the GSK3β site (Thr509/Thr514) of CRMP2 (Varrin-Doyer et al., 2009). CXCL12/SDF1 also induces tyrosine phosphorylation at Tyr479 *via* Yes kinase, and the introduction of CRMP2-Tyr479Phe expression suppresses the migration of Jurkat cells, indicating the importance of tyrosine phosphorylation of CRMP2 (Varrin-Doyer et al., 2009).

## CRMP2-Interacting Drugs

Several small molecules that have been shown to bind CRMP2 are lacosamide (Wilson et al., 2012, 2014), lanthionine ketimine (LK; Hensley et al., 2010a, 2013; Hensley and Harris-White, 2015), edonerpic maleate (Abe et al., 2018) and naringenin (Ghofrani et al., 2015; Lawal et al., 2018). These molecules are considered candidates for application in the treatment of some of the CRMP2-related pathological conditions discussed above.

(R)-Lacosamide inhibits CRMP2-mediated neurite outgrowth in cultured cortical neurons. (R)-lacosamide reduces CRMP2-mediated tubulin polymerization *in vitro* (Wilson et al., 2012). It seems to prevent posttraumatic axon sprouting *in vivo*. An *in vitro* study showed that (S)-lacosamide, a stereoisomer of the clinically used antiepileptic drug (R)-lacosamide, impairs the ability of CRMP2 to enhance tubulin polymerization *in vitro* without altering tubulin-binding (Wilson et al., 2014).

Lanthionine ketamine-ethyl ester (LKE) is a synthetic cell-penetrating ester derivative of LK, an endogenous sulfur amino acid metabolite in the mammalian brain (Hensley et al., 2010a). LK and LKE bind directly to CRMP2 and change the binding affinity of CRMP2 to its binding partners such as tubulin dimers and neurofilaments. LKE administration reduces CRMP2-tubulin affinity while enhancing CRMP2-neurofilament binding. The neurite growth-promoting action of LKE has been reported in NSC-34 mouse motor neuron-like cells and primary chick DRG neurons (Hensley et al., 2010a). LKE has also been shown to have a neuroprotective effect on these cells from oxidative stress insults. When applied to the SOD1^G93A^ transgenic mouse model of ALS, LKE was reported to delay progressive neurodegeneration (Hensley et al., 2010b). LKE treatment also substantially diminished cognitive decline and brain amyloid-β (Aβ) peptide deposition and phospho-tau accumulation in the 3 × Tg-AD mouse model of AD, reducing the density of Iba1-positive microglia (Hensley et al., 2013). In this study, LKE normalized CRMP2 phosphorylation at Thr514 and suppressed neuroinflammation in the brains of these mice (Hensley et al., 2013). LKE has also been tested in a mouse model of SCI and was reported to benefit the recovery of motor function and reduce post-traumatic neuroinflammation (Kotaka et al., 2017).

Edonerpic maleate has been shown to bind CRMP2, and facilitate experience-driven synaptic glutamate α-amino-3-hydroxy-5-methyl-4-isoxazole-propionic-acid (AMPA) receptor delivery and accelerate motor function recovery after motor cortex cryoinjury in mice in a training-dependent manner through cortical reorganization (Abe et al., 2018). Edonerpic maleate decreased the amount of phosphorylated form of CRMP2, and activated actin-depolymerizing factor (ADF)/cofilin, thereby leading to the trafficking of AMPA receptors into the spine surface under plasticity-inducing conditions. The drug failed to augment recovery in CRMP2-deficient mice, suggesting a CRMP2-dependent action (Abe et al., 2018).

Naringenin has demonstrated the ability to bind and decrease CRMP2 phosphorylation (Yang et al., 2016; Lawal et al., 2018). This action of changing CRMP2’s phosphorylated status and hence the binding of cytoskeletal elements may account for the ability of naringenin to improve AD pathology and cognitive deficits in mouse models of AD. For example, naringenin has been shown to significantly improve the performance of Aβ-injected rats in passive avoidance and radial arm maze tasks (Ghofrani et al., 2015). In a 5xFAD mouse model of AD, naringenin ameliorated memory deficits and decreased amyloid plaques and phosphorylated tau (p-tau; Yang et al., 2016). Some of the beneficial effects of naringenin in rodent models of AD may also be related to its anti-inflammatory actions (Park et al., 2012; Wu et al., 2016).

## Conclusion

The CRMPs family of proteins appear to coordinately be involved in several coordinated biological events including axon guidance, target recognition, synapse maturation, and dendritic branching. The CRMPs possess the ability to interact with various kinds of molecules, thereby being involved in these many processes. Although the CRMPs themselves are regulated by versatile enzymes having a wide substrate spectrum, successful chemical modulators or therapeutic agents may be developed to act on such protein-protein interaction interfaces.

## Author Contributions

FN, TO, and YG contributed to the writing and editing of this review.

## Conflict of Interest

The authors declare that the research was conducted in the absence of any commercial or financial relationships that could be construed as a potential conflict of interest.
